# Towards MR-guided EP interventions using an RF-safe approach

**DOI:** 10.1186/1532-429X-11-S1-O84

**Published:** 2009-01-28

**Authors:** Sascha Krueger, Oliver Lips, Bernd David, Daniel Wirtz, Steffen Weiss, Steen F Pedersen, Dennis Caulfield, Julian Bostock, Reza Razavi, Tobias Schaeffter

**Affiliations:** 1 Philips Research Europe, Hamburg, Germany; 2 MR Research Centre, University Hospital, Aarhus, Denmark; 3 Division of Imaging Sciences, King's College, London, UK

**Keywords:** Catheter, Fiber Optic Temperature, Intracardiac Electrogram, Prototype Setup, Tracking Coil

## Introduction

Various cardiac arrhythmias, e.g. atrial fibrillation and ventricular tachycardia, can be treated by electrophysiological (EP) interventions [1]. Applying MR for guiding these interventions offers advantages like 3D visualization of the cardiac soft tissue in relation to the catheter and absence of ionizing radiation [2]. In this work, a prototype MR-EP system and catheter for diagnostic EP-interventions is described, which integrates concepts for RF-safe MR-tracking [3] and EP diagnostics [4]. The operation of the system is demonstrated in MR-guided EP experiments in pigs including mapping and pacing. RF-safety of the diagnostic MR-EP catheter prototype is shown and signal quality is compared to conventional EP catheters.

## Materials and methods

### System setup

All experiments were performed on a clinical whole-body 1.5 T MR scanner (Achieva I/T, Philips Healthcare, Netherlands) equipped with an in-room display and an additional MR-EP-workstation (MR-EP-WS) including a standard EP-recorder (EP Tracer, CardioTek, Netherlands). This workstation, located next to the scanner, combines and displays incoming real-time 2D and 3D images and real-time tracking positions from the MR scanner as well as real-time EP-data from the EP-recorder.

**Figure 1:**
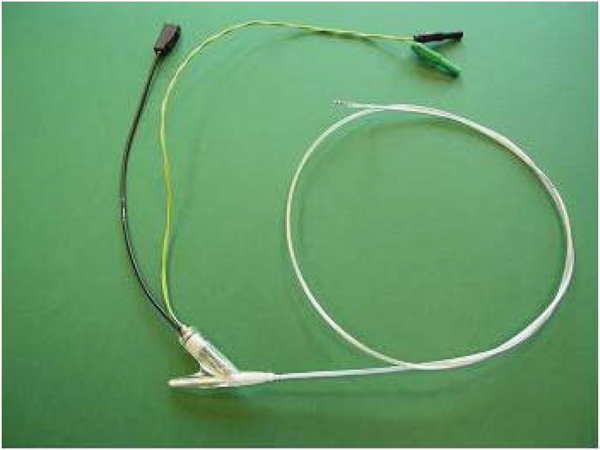
**Diagnostic RF-safe MR-EP catheter**.

A 7F diagnostic EP catheter (Fig. 1) with two ring electrodes and a tracking coil was used. Intracardiac and tracking signals are transferred via RF-safe high resistance wires [2] and a transformer-based transmission line [3], respectively.

### Comparison MR-EP/conventional EP catheter

Conventional diagnostic EP catheters (Supreme Quad, JSN, 5F, St Jude, MN) and MR-EP catheters were compared under X-ray. Bipolar intracardiac electrograms (IEGM) were acquired with both catheters at corresponding locations (RA lateral wall, RV apex, TV ring, and HIS).

### In-vivo proof of RF-safety

Temperature recordings during a typical real-time bFFE sequence (TR 2.4 ms, flip 65°, global SAR 4 W/kg) were performed for the MR-EP and the conventional EP catheter. The catheters were equipped with fiber optic temperature probes and were inserted into the RA.

#### EP-Mapping procedure

The RA and RV were mapped using the MR-EP system and catheters. 3D bFFE and 3D CE-MRA datasets were acquired prior to catheterization of the animals. All MR and EP data can be combined and displayed on the MR-EP-WS for guidance, including a surface model of the cardiac vessels, reformatted slices at the catheter position either manually angulated or using the real-time MR imaging geometry.

## Results

### Comparison with conventional EP catheter

**Figure 2:**
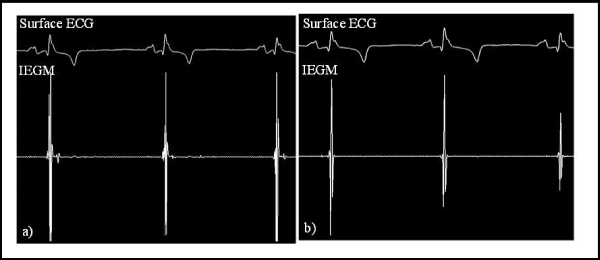
**EP signals acquired in the RV (a) with the MR-EP catheter equipped with highly resistive wires and (b) with the conventional catheter**.

IEGMs acquired with the MR-EP catheter were equivalent in quality to those acquired with the conventional EP catheter (Fig. 2).

### In-vivo proof of RF-safety

**Figure 3:**
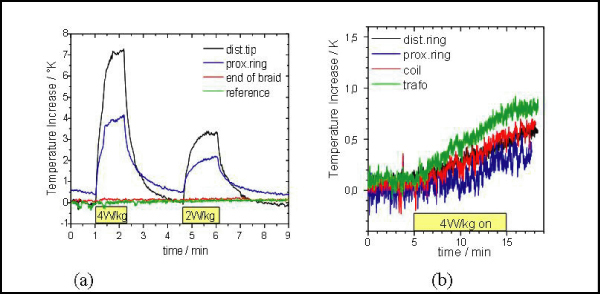
**In-vivo temperature increase (a) with the MR-EP catheter and (b) with the conventional catheter**.

The MR-EP catheter's maximal temperature increase after 10 min of RF transmission at 4 W/kg was 0.7 K (Fig. 3a) almost corresponding to the expected increase in global body temperature (0.6 K). Hence, device-related local heating effects are negligible.

In contrast, an increase of up to 7.5 K in only 80 s was observed at the tip of the conventional catheter (Fig. 3b).

### EP recording under MRI

**Figure 4:**
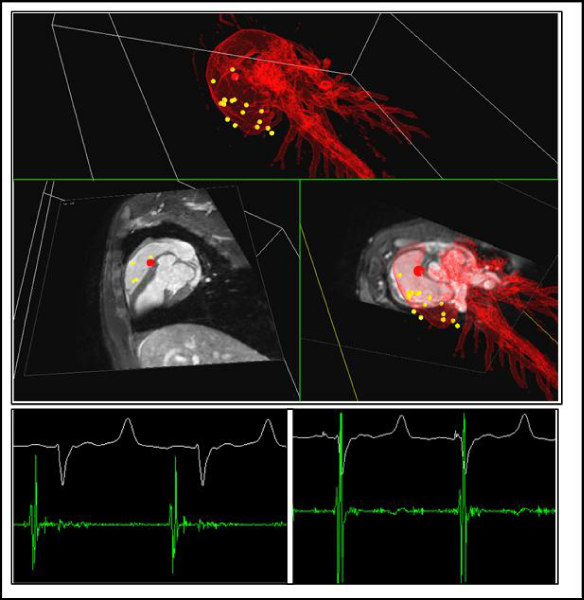
***Top*** **: Roadmap-based real-time 3D-visualization of the catheter position during recording (red dot) on the MR-EP workstation**. The yellow dots in the 3D rendering of the heart indicate previous mapping positions. *Bottom*: In-bore EP recordings at two selected positions showing an atrial signal (left) and a ventricular signal (right).

The MR-EP-WS enabled a fast mapping, e.g. 40 points in RV in 20 min. The in-bore IEGM recordings were comparable to those under X-ray (Fig. 4).

Furthermore, atrial and ventricular pacing was achieved via the MR-EP catheters. Successful stimulation was confirmed by a second MR-EP catheter and was also clearly visible in the surface ECG.

## Conclusion

Recording of intracardiac electrograms is feasible with the MR-EP catheter. EP data quality is equivalent to conventional EP catheters. The combined use of highly resistive wires and a transformer-based transmission line for active tip tracking effectively suppresses RF-heating even during high SAR MRI.

The prototype setup of the MR-EP system provided excellent guidance and an efficient workflow for diagnostic MR-EP interventions.
